# “In Limbo”—use of, and alterations to, modified diets by nursing home staff in the absence of timely specialist support

**DOI:** 10.3389/fresc.2024.1276713

**Published:** 2024-02-16

**Authors:** Mary Okon, Kei Yen Chan, Shaun T. O’Keeffe

**Affiliations:** Department of Geriatric Medicine, Galway University Hospital, Galway, Ireland

**Keywords:** dysphagia, nursing homes, modified diets, thickened liquids, informed consent

## Abstract

**Background:**

Dysphagia is common in nursing home (NH) residents. Staff may not always be able to access speech and language therapist (SLT) assessments in a timely manner and there are some reports of nurses initiating or changing modified diets in these circumstances.

**Methods:**

A mixed quantitative and qualitative approach was used to analyse responses to an online anonymized survey of senior nurses working in Irish NHs. They were asked about their experience of delays accessing SLT services and whether they would ever initiate or change modified diets. Respondents were asked if they would give water to a thirsty resident, prescribed mildly thick liquids, who demanded it on a hot day because thickened fluid was not thirst quenching.

**Results:**

Of 77 nurses surveyed, 63 (82%) responded. Three quarters reported delays accessing SLT services sometimes or often. Thirty-four (54.0%) would not give the thirsty resident water. About 70% reported that thickened fluids or modified texture diets were started without SLT sometimes or often. A third of respondents would thicken fluids or modify food to a greater extent than previously recommended but very few would make a diet less restrictive. The main themes that emerged from the comments provided were related to the uncertainty and dilemmas created for staff, what mitigating actions they might take in those circumstances and the need for better guidance and better access to SLT services.

**Discussion:**

Delays accessing SLT services are common for Irish NHs, and staff may initiate or change modified diets themselves in these circumstances. The responses suggest a widespread, and unjustified, belief that thicker or more modified is better for those with dysphagia. Clear and accurate guidance, and a better SLT service, is needed for NH staff.

## Introduction

Oropharyngeal dysphagia is common in nursing home (NH) residents, many of whom have neurological or neurodegenerative conditions. There is evidence from Ireland and elsewhere that nursing home (NH) staff who have concerns about residents' abilities to eat drink and swallow safely may be unable to access specialist speech and language therapist (SLT) assessments in a timely manner (1–4). A survey conducted by Nursing Homes Ireland noted that some had to rely on SLTs provided by nutritional companies or to ask residents to pay for private assessments (2).

Some reports also suggest that, in the absence of SLT support, nurses may sometimes themselves initiate or change modified diets (modified texture food and thickened liquids) for residents with dysphagia (5–8). In this study we sought to determine the approach of Irish NH nursing staff in these circumstances.

## Methods

Senior nurses working in NHs in Ireland attending educational webinars (none was focused on dysphagia) were invited to participate in an online anonymized survey. In addition to the choices offered for questions, participants were encouraged to provide comments or to email the authors afterwards. Comments and responses were analysed using a thematic analytic approach (9).

Demographic details about the nursing homes and the estimated proportion of residents receiving modified diets were collected. Participants were asked about delays encountered accessing SLT services (all such questions had the options of never, sometimes, often or always).

Participants were presented with the following vignette: “*Joe is 72 and was admitted to your unit following a stroke three months ago. He is confined to a wheelchair and needs staff to provide food and drink which he consumes himself. He was discharged from hospital on Level 2* (referring to the International Dysphagia Diet Standardisation Initiative (IDDSI) Framework (10) *(mildly thick) liquids. He has mild aphasia but can express himself clearly. He is generally well and medically stable. It is now a very warm weekend day, and you are the senior nurse on duty. John says he is very thirsty, and the thickened fluids don't relieve his thirst. He demands that staff give him ordinary (unthickened) water”.* They were asked to say whether or not they would give Joe water, asked to explain their reasoning and whether they had encountered such cases.

Participants were asked how often, in their experience, patients on a diet initially recommended by an SLT were subsequently reviewed. Finally, they were asked a series of questions about initiating or changing a dietary recommendation for residents with suspected difficulty swallowing.

## Results

There were attendees from 77 NHs (51 private or voluntary) at the webinars, and 63 (82%) responded to the online survey. Median [interquartile range (IQR)] number of residents was 56 (40–78). Median (IQR) percentage residents taking thickened fluids and modified texture diets respectively were 20 (16–29)% and 29 (24–35)%. Over three quarter of responds reported that delays accessing SLT services occurred sometimes or often in their facility and that review by an SLT of a previously recommended diet occurred never or sometimes (Table 1).

**Table 1:** Responses to survey questions.

Questions	Responses (*N *= 68)				
	Never	Sometimes	Often	Always	No response
Does your facility encounter delays in accessing SLT services for residents?	2 (3.2%)	30 (47.6%)	19 (30.2%)	12 (20.6%)	0 (0%)
Are residents on a diet initially recommended by an SLT subsequently reviewed by an SLT in your experience?	35 (55.6%)	13 (20.6%)	7 (11.1%)	0 (0%)	8 (12.7%)
How often would you start thickened fluids without an SLT seeing the resident?	10 (15.9%)	35 (55.6%)	8 (12.7%)	0 (0%)	10 (15.9%)
How often would you start a modified food diet without an SLT seeing the resident?	2 (3.2%)	25 (39.7%)	19 (30.2%)	7 (11.1%)	10 (15.9%)
	Yes	No	No response		
Would you give Joe (see vignette in text) unthickened water?	19 (30.2%)	34 (54.0%)	10 (15.9%)		
Have you ever encountered a similar situation to that presented in the vignette?	30 (47.6%)	19 (30.2%)	14 (22.2%)		
Would you ever thicken fluids more than initially recommended by an SLT?	20 (31.7%)	30 (47.6%)	13 (20.6%)		
Would you ever thicken fluids less than that initially recommended by an SLT?	4 (6.4%)	46 (73.0%)	13 (20.6%)		
Would you ever modify a food diet more than initially recommended (e.g. puree instead of minced and moist)?	22 (33.3%)	30 (47.6%)	11 (17.5%)		
Would you ever modify a food diet less than that initially recommended (e.g. minced and moist instead of pureed)?	2 (3.2%)	46 (73.0%)	15 (23.8%)		

Regarding the vignette, 19 (30.2%) would give Joe unthickened water, 34 (54.0%) would not and 10 (15.9%) did not provide a yes/no answer and almost half the respondents had encountered similar cases in their practice.

Most respondents would start thickened fluids or a modified food diet without an SLT seeing the resident sometimes or often. The most common level of thickened flids commenced this way were “mildly” or “very mildly” thickened although there were comments about the “usual” or “standard” amount of thickener. The most common level of modified diet commenced was a soft and bite sized diet, but 14 (22.2%) respondents said that food might be pureed. Almost a third of respondents would thicken fluids or modify food diets fluids more than initially recommended, but very few would make fluid less thick or food less modified than recommended by an SLT.

The main themes that emerged from the comments provided were related to the uncertainty and dilemmas created for staff, what mitigating actions they might take in those circumstances and the need for better guidance and better access to SLT services (Table 2). There were many comments suggesting that nurses are “caught in the middle” or “in limbo” when they can't access SLT services quickly. Some suggested that Ireland's new Assisted Decision-Making Act, for those whose decision-making capacity is in question (11), with its emphasis on the “will and preference” of the person and an (inaccurately) alleged “right to make unwise decisions”, was making things harder for staff faced with such dilemmas.

**Table 2:** Main themes on analysis of comments from respondents.

Uncertainty and dilemmas for staff • Respect for autonomy and choice vs. Risks for residents • Competing risks for residents: aspiration risk vs. poor intake if dislikes fluid and food modifications • Legal issues—decision-making capacity legislation • Professional responsibilities—duty of care vs. acting beyond accepted scope of practice
Trying to resolve uncertainty and dilemmas • Asking for SLT assessment or review • Careful documentation by staff • Asking resident to sign a consent form or waiver • Asking doctor, family or next of kin to approve • Asking for a decision making capacity assessment
Need for improvements • Need for better guidance and education for staff • Need for improved access to SLT services

Of those who would give Joe water, many noted that it would be given slowly and in small amounts to check it wasn't “going down the wrong way”. Several commented that he seemed to have capacity to decide and that it was his choice, even if potentially unwise. There were also comments on the need to document their advice that he should have thickened fluids or of asking him to sign an ad-hoc consent form.

Most comments from those who wouldn't give Joe water or didn't give a yes/no response related to the perceived high risk that he would aspirate and get pneumonia. Some were concerned about their legal responsibility, about possible disciplinary action and about breach of their “duty of care”. Before he could receive water, different respondents said he would need SLT reassessment, approval from his doctor, family or “next of kin” or an assessment of his decision-making capacity.

## Discussion

A high proportion of residents in this study were reported to receive thickened fluids and modified texture diets, which is consistent with many other reports (12–14). Our results agree with those from a previous survey of Irish staff which concluded that “dysphagia management services are currently inadequate in Irish NHs” (1). Delays accessing SLT services were common, and reviews by SLTs of previously recommended diets were uncommon (15).

This study confirms reports from other countries that nurse-initiated dietary restrictions and changes are not unusual, and that the most common approach is to introduce a more restrictive diet such as thicker liquids or pureed food (4–8). Sometimes this reflects difficulty accessing SLT services. Some studies of nursing home staff reported that, despite a lack of training, nurses felt they had the skills to assess swallowing problems and to make appropriate recommendations (7, 8).

Initiating some modification of food texture is often the right thing to do when people have difficulty chewing and swallowing. It is important, for example, to cut food into bite sized chunks for those at risk of asphyxiation who can't do so themselves. However, there are widespread beliefs that “thicker is safer” for liquids and that pureed food is the safest option in those with dysphagia (13, 16). These are reflected in the responses to our vignette where most respondents wouldn't “allow” Joe unthickened water in case he aspirated and got pneumonia (17). They also underlie the recent vogue for “risk feeding” policies (18).

Although dysphagia increases the risk of aspiration of food and fluid and is associated with a greater risk of pneumonia, there is no simple linear relationship between aspiration and pneumonia. There is no good evidence that modified diets do reduce pneumonia in people with dysphagia (13, 19, 20). Use of thickened liquids is associated with reduced fluid intake, and texture-modified foods contribute to undernutrition (14, 20, 21). This is a particular concern when many NH residents are already underhydrated and undernourished (22). Most importantly, modified diets can further worsen the quality of life of those with dysphagia (23).

Although current practice is suboptimal, we are sympathetic towards NH staff faced with these issues. They are indeed, as their comments note, in an invidious position when they have concerns about the safety of swallowing and no ready access to specialist advice. Their concerns about being criticized for adverse outcomes are genuine (17, 18). Surveys of practicing SLTs show a dissociation between the paucity of evidence supporting recommendations for modified diets and their attitudes, beliefs and practices (16). This will inevitably influence nurses, catering, and other staff in NHs and elsewhere. It is essential that SLT recommendations and their communications with NH staff (and patients) reflect the evidence regarding modified diets. This is even more important if review is not planned or is not possible.

A limitation of our study is that it was restricted to one country and the number of respondents was small. However, the responses in this study are consistent with those reported from other developed countries. The average number of residents in the NHs represented and the mix of public and private facilities in this study was close to the Irish national average (2).

We have previously argued that recommendations around dietary modifications should be seen through the “lens” of informed consent and a need for true shared decision making (19). Accurate communication with residents requires professionals to have a good understanding of the issues involved. It is important that NH staff are provided with clear and accurate guidance, and access to timely advice, when they have concerns that residents have difficulty eating, drinking and swallowing.

## Data Availability

The original contributions presented in the study are included in the article/Supplementary Materials, further inquiries can be directed to SO'K, sokeeffeanc@gmail.com.
